# Supplementary material for: Practical Application of a Urinary Zearalenone Monitoring System for Feed Hygiene Management of a Japanese Black Cattle Breeding Herd—Relevance to Anti-Müllerian Hormone and Serum Amyloid A Clarified from a Two-Year Survey
Source: Toxins (Basel). 2023 Apr 30;15(5):317. doi: 10.3390/toxins15050317 (PMC10224239; doi:10.3390/toxins15050317)
Supplement: Supplementary file 1 [file toxins-15-00317-s001.zip › toxins-2358726-supplementary.pdf]

Practical Application of a Urinary Zearalenone Monitoring System for Feed Hygiene Management of a Japanese Black Cattle Breeding Herd—Relevance to Anti-Müllerian Hormone and Serum Amyloid A Clarified from a Two-Year Survey

Oky Setyo Widodo, Dhidhi Pambudi, Makoto Etoh, Emiko Kokushi, Seiichi Uno, Osamu Yamato, Masayasu Taniguchi, Mirni Lamid and Mitsuhiro Takagi

Table S1. The raw data of ZEN, AMH, and SAA measurement results.

Date	Cow numbers (ear tag number)																	
	1 (0842)			2 (2291)			3 (2646)			4 (2899)			5 (4622)			6 (6074)		
	ZEN	AMH	SAA	ZEN	AMH	SAA	ZEN	AMH	SAA	ZEN	AMH	SAA	ZEN	AMH	SAA	ZEN	AMH	SAA
July, 2020	2257.7	1230	5.1	1382.5	1357	3.3	4366	1290	1.8	2445.4	1241	2.1	1353.7	2490	2.5			
August, 2020	9021.1	1247	2.9	5802.3	674	23.4	9662.3	1441	2.1				5222.3	2099	2.2			
September, 2020	335.5	1506	2.2	324.9	870	3.6	668.1	1445	1.9	915.8	937	5.7	576.1	2032	2.3			
October, 2020	1585.1	1219	2.1	800.8	1093	18.1	946.2	1294	2.3	925.4	1252	4.5	1236.3	1925	2.5			
November, 2020	586.5	954	3.1	618.2	1853	3.1	609.8	1331	5	803.4	1481	2.5	471.4	1465	3.2			
December, 2020	1146.9	1338	4.7	467.2	2429	3.5	590.5	1548	2.7	1616.4	1696	2.3	380.3	1318	2.6			
January, 2021	699.4	1759	6.1	520.3	2639	26.8	884.2	1513	2.5	936.5	1890	2.4	471.5	1303	2.9			
February, 2021	1284.8	1979	5.5	886.8	1985	3.3	730.9	1563	2.2	1678.9	1918	2.3	631.3	744	2.9			
March, 2021	712.8	1864	5.5	618.2	1950	19.7	924.6	1073	4.9	769.0	2140	2.7	520.0	1351	3.1			
April, 2021	3866.8	1808	3.4	1604.4	1377	3.8	3784.0	1185	4.7	9710.6	2099	2.3	1460.1	1300	2.8			
May, 2021	1178.7	1931	2.8	1899.8	1221	2.7	2500.3	1399	1.1	3589.3	1910	1.9	849.1	2061	14.5			
June, 2021	1814.0	1552	3.0	763.6	964	7.4	2012.2	1564	1.6	1785.8	1600	1.8	782.4	1505	2.0			
July, 2021	1534.5	1827	1.7	2397.7	886	3.1	2945.7	2174	1.2	3237.6	1466	2.1	1650.8	1902	1.3			
August, 2021	1370.8	1635	1.2	1434.8	933	17.4	2901.0	2015	8.1	2738.7	1097	4.1	1207.2	1599	1.0			
September, 2021	557.1	663	20.3	831.0	938	2.7	2778.2	1727	1.5	4948.0	1105	13.3	2752.4	984	7.5			
October, 2021	313.9	921	3.2	641.3	1970	5	732.4	1911	1.7	860.8	1253	35.1	565.9	1271	1.7			
November, 2021	910.4	1134	5.7	576.4	2262	3.1	502.9	1232	2.3	957.8	734	6.7	425.0	511	1.7			
December, 2021	518.8	1211	4.5	830.9	2140	0.5	722.9	1456	0.8	1132.9	1499	17	604.8	777	2.9			

January, 2022	711.2	1767	0.2	361.9	2157	0.1	491.4	1248	0	545.9	1685	0	558.7	713	4.6			
February, 2022	2410.4	1731	6.9		1504		1224.6	969	3.5	1334.9	1443	2.1	661.4	816	3.3	709.2	285	4.5
March, 2022	697.1	1851	0.6	889.5	1725	0.6	848.6	709	7				266.8	1332	0.4	509.6	280	0.5
April, 2022	627.2	2059	3.9	746.4	1451	5.2	1312.8	923	6.3				566.9	1423	2.8	245.1	407	8.2
May, 2022	514.7	1622	2.3	904.0	1082	1.1	815.3	1003	4.3				383.7	1964	0.5	684.4	635	1.0
June, 2022	995.8	1374	1.0	453.5	982	2.2	1166.2	1625	0.7				409.8	1766	10.9	610.2	528	1.3
July, 2022	333.3	1680	0.9		569	23.0	461.0	2075	0.9				438.4	2196	1.3	415.9	671	1.6

ZEN (pg/mg Cre); AMH (pg/mL); SAA (mg/L).
Cow 6 is a replacement cow from the same herd after Cow 4 was culled; Sampling could not be performed for Cow 4 in August 2020 because she was about to calve at the time of sampling; ZEN and SAA Cow 2 were not obtained in February 2022, nor was ZEN in July 2022.
